# The difference in local, regional and distant breast cancer recurrence between the immediate and delayed DIEP flap procedure; a retrospective cohort study

**DOI:** 10.1007/s10549-021-06199-3

**Published:** 2021-05-24

**Authors:** M. E. M. Joosen, S. J. Schop, L. L. Reinhoudt, S. M. J. van Kuijk, J. Beugels, A. P. de Bruïne, D. Goudkade, E. M. Heuts, R. R. W. J. van der Hulst, A. A. Piatkowski de Grzymala

**Affiliations:** 1 Department of General Surgery, Maastricht University Medical Center+, P. Debyelaan 25, 6229 HX Maastricht, The Netherlands; 2 Department of Plastic Surgery, Maastricht University Medical Center +, P. Debyelaan 25, 6229 HX Maastricht, The Netherlands; 3 Department of Clinical Epidemiology and Medical Technology Assessment, Maastricht University Medical Center+, P. Debyelaan 25, 6229 HX Maastricht, The Netherlands; 4 Department of Pathology, VieCuri Medical Center, Tegelseweg 210, 5912 BL Venlo, The Netherlands; 5 Department of Pathology, Zuyderland Medical Center Sittard-Geleen, Dr. H. van der Hoffplein 1, 6162 BG Sittard-Geleen, The Netherlands

**Keywords:** Breast cancer, Breast reconstruction, Reconstructive surgery, Autologous breast reconstruction, DIEP flap procedure, Oncological safety

## Abstract

**Purpose:**

It has been hypothesized that autologous breast reconstruction can cause reactivation of dormant micro metastases by its extensive tissue trauma, influencing the risk of breast cancer recurrence. However, about the specific effect of timing on breast cancer recurrence in the deep inferior epigastric perforator (DIEP) flap reconstruction is not much known. In this study the rate of local, regional and distant recurrence between patients undergoing an immediate and delayed autologous DIEP flap breast reconstruction were evaluated.

**Methods:**

In this retrospective cohort study, breast cancer patients undergoing a DIEP flap breast reconstruction between 2010 and 2018 in three hospitals in the Netherlands were evaluated. Cox proportional hazards regression analyses were performed to assess the impact of different factors on breast cancer recurrence. The primary endpoint was local breast cancer recurrence. Secondary endpoints were regional and distant recurrence.

**Results:**

A total of 919 DIEP-flap reconstructions were done in 862 women of which 347 were immediate- and 572 were delayed DIEP flap reconstructions. After a median follow-up of 46 months and 86 months respectively (*p* < 0.001), local breast cancer recurrence occurred in 1.5% and in 1.7% of the patients resulting in an adjusted hazard ratio of 2.890 (*p* = 0.001, 95% CI 1.536, 5437).

**Conclusion:**

This study suggests an increased risk for breast cancer recurrence in women receiving a delayed DIEP flap reconstruction as compared to women receiving an immediate DIEP flap reconstruction. However, these data should be interpreted carefully as a result of selection bias.

**Supplementary Information:**

The online version contains supplementary material available at 10.1007/s10549-021-06199-3.

## Introduction

Breast cancer survival has substantially improved over the past decades [1, 2]. Still, due to the advent of genetic testing and population screening, mastectomy rates typically range from 25 to 40% [3]. Studies have shown that breast reconstruction improves the quality of life and body image for its positive effects on sexual and psychosocial wellbeing [4, 5]. Therefore a rising number of women opt for breast reconstructive surgery after mastectomy.

Currently, the deep inferior epigastric perforator (DIEP) flap is the first choice for autologous breast reconstruction [6]. This reconstruction can be either performed immediately following the mastectomy or in a delayed setting. Immediate reconstruction results in better aesthetic outcome due to skin-sparing treatment, and less psychological distress. Still, the delayed DIEP flap procedure is performed more frequently [7].

A recent study showed that there is no statistically significant difference between the immediate DIEP and the delayed DIEP group when it comes to major complications [8]. However, there is not much known about the oncological safety of autologous breast reconstruction itself, specifically the DIEP flap. It has been suggested that severe surgical trauma triggers tumor growth in patients with previous breast cancer [9]. A potential explanation might be tumor dormancy in which dormant circulating tumor cells, originating from micro metastases, remain dormant until they are either reactivated by an enhancing effect of surgery or eliminated. The surgical caused transition may be initiated by releasing angiogenic agonists, immunomodulating factors and growth factors [10, 11]. The extensive and prolonged autologous breast reconstructive surgery may be considered a major tissue trauma suggesting autologous breast reconstruction increases the breast cancer recurrence risk. However, studies have shown different results [12–15], probably owing to inclusion of heterogenous breast reconstructive surgery and lack of homogeneous study populations.

Moreover, most studies evaluated the recurrence rates in delayed DIEP flap surgery compared to mastectomy alone. To the best of our knowledge, no research has been published on the specific effect of timing (immediate or delayed) on breast cancer recurrence in DIEP flap reconstruction surgery in a clinical setting. The aim of this study was to analyse the influence of timing, on the recurrence rate, by comparing the local, regional, and distant recurrence rates of patients who received immediate DIEP flap reconstruction to the recurrence rates of patients who received delayed DIEP flap reconstruction.

## Methods

In this multicenter retrospective cohort study, the data of all patients with a history of breast cancer who underwent an immediate and delayed DIEP flap breast reconstruction between January 2010 and December 2018 at Maastricht University Medical Center (MUMC +) and two community hospitals (VieCuri Medical Center Venlo and Zuyderland Medical Center Sittard) were reviewed. The study was approved by the medical ethics committee and was performed in accordance with the ethical standards of the Declaration of Helsinki.

All surgeries were performed by a group of ten plastic surgeons with variable experience in microsurgery. The majority of the surgeons operated in both university hospital and at least one of the community hospitals. More complex patients were usually referred to the university hospital. With the exception of three bilateral procedures, all bilateral DIEP flap breast reconstructions were performed at the university hospital.

All participants were female, older than 18 years and had a diagnosis of breast cancer in the breast to be reconstructed. Reconstructions after prophylactic mastectomy were excluded, as well as women who had a metastasis at the time of mastectomy. Women who had a delayed reconstruction and experienced a recurrence after mastectomy, but before reconstructive surgery, were excluded as well. These women were excluded, because this study aims to determine the association between timing of reconstruction surgery and first recurrence and these women were no longer a risk for a first recurrence.

DIEP flap reconstruction was dichotomized into ‘immediate reconstruction’ and ‘delayed reconstruction’; immediate reconstruction being defined as reconstruction in the same operation as mastectomy, while delayed reconstruction implied a secondary surgery after the mastectomy at any other time point. Medical records were reviewed and data were collected. Patient data included patient demographics, type of reconstruction (unilateral or bilateral), side, TNM stage, histological type of breast cancer, axillary lymph node status, estrogen receptor (ER) status, progesterone receptor (PR) status, human epidermal growth factor receptor 2 (HER2) amplification status and type of therapy (neoadjuvant and/or adjuvant). The primary endpoint was local breast cancer recurrence. Secondary endpoints were regional and distant recurrence. The length of follow-up was considered as the time between the date of mastectomy until the last date of follow-up or the first recurrence or death from another cause than breast cancer. Patients who underwent any other autologous reconstruction or had flap loss were excluded.

Municipal basic administration was used to check whether patients were deceased during follow-up. Finally, pathological information was checked twice, both in medical records and in PALGA (National pathology archive).

In patients who received breast conserving surgery (BCS) before (prophylactic) mastectomy, the pathological tumor size and the histological type of tumor of the BCS was used. In all other patients the pathological tumor size and the histological type of tumor after mastectomy was used.

Histologically confirmed breast cancer recurrence in the chest wall, ipsilateral skin, or within or adjacent to the transposed abdominal tissue was defined as local recurrence. Regional recurrence was considered as recurrence in the following lymph nodes: supraclavicular or infraclavicular, ipsilateral or contralateral axillary, and internal or interpectoral mammary. Recurrence at any other site was considered distant recurrence. Every recurrence, either confirmed by histopathology or cytology, was recorded according to its date of diagnosis.

### Statistical analyses

In case of incomplete patient records, data were imputed using multiple imputation with fully conditional specification. The number of imputations was set to 5, and imputations were drawn using predictive mean matching. Baseline characteristics were summarized in detail: continuous variables as mean or median. Categorical variables were presented as percentages and numbers. The means of continuous outcome variables were compared between the immediate and delayed groups, using the independent samples *t* test. To compare the distribution of categorical variables between the cohorts we used the Chi-square test.

The primary unit of analysis was the patient—rather than the flap—for recurrence analyses. Even though in a bilateral breast reconstruction there are two flaps and therefore hypothetically more risk, there is no evidence a single flap, independent of patient characteristics, can cause recurrence.

Cox proportional hazards regression analysis was performed to assess the association between timing of breast reconstruction and recurrence. The proportional hazards assumption was verified using visual inspection of the Schoenfield residuals. First, we performed a naive analysis ignoring the time of delayed reconstruction. Afterwards, delayed reconstruction (e.g. time between mastectomy and reconstruction surgery) was entered into the model as a time-varying covariate to account for immortal time bias. This bias arises when the period between cohort entry and date of first exposure to a treatment, during which recurrence or death has not occurred, is either misclassified or simply excluded.

Potential confounders, including demographic characteristics and disease-specific characteristics, were added to a multivariable Cox regression. As the majority of women received unilateral surgery, we did not employ multilevel modelling to take clustering of observations within women into account.

In contrast to the analysis of local and regional recurrences, in situ carcinomas were excluded in the analysis of distant recurrence.

Hazard ratios (HR) were computed to present associations, accompanied by 95% confidence intervals. Statistical significance was considered at *p* < 0.05. All statistical analyses were performed using IBM SPPS Statistics version 26 for Windows.

## Results

### Patient demographics and tumor characteristics

**Fig. 1:**
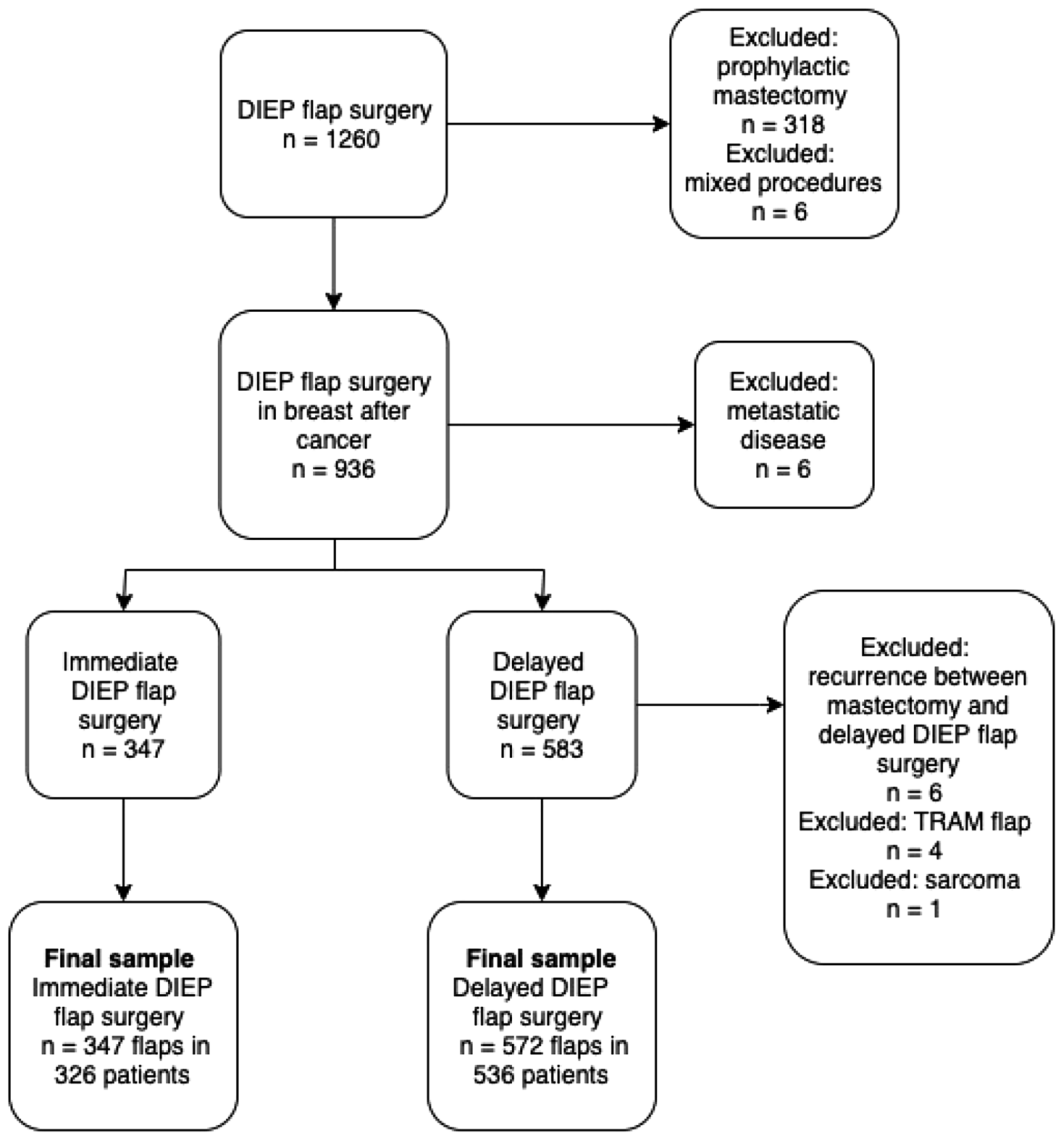
Flow chart of patient inclusion. TRAM; transverse rectus abdominis myocutaneous

Between January and December 2018, a total of 1260 DIEP flap reconstructions were performed in 971 patients. After excluding mixed bilateral procedures, prophylactic mastectomies, recurrence before reconstruction and a metastasis at the time of mastectomy, 919 DIEP flap breast reconstructions (*n* = 347 immediate and *n* = 572 delayed reconstructions) in 862 patients were included. The flow chart of patient inclusion is presented in Fig. 1.

In Maastricht University Medical Center, 536 flaps (58.3%; *n* = 216 immediate and *n* = 320 delayed) were performed in 480 patients; in VieCuri Medical Center, 198 flaps (21.5%; *n* = 119 immediate and *n* = 79 delayed) were performed in 198 patients; and in Zuyderland Medical Center, 185 flaps (20.1%; *n* = 12 immediate and *n* = 173 delayed) were performed in 184 patients. Median follow-up was 46 months (IQR 45) and 86 months (IQR 54.75 months) in the immediate and delayed reconstruction group, respectively.

Table 1 is a description of demographic data and oncological treatment in the immediate and delayed reconstruction group. An overview of the tumor characteristics is provided in Table 2. Patients in the immediate group significantly more often had a bilateral DIEP flap reconstruction (*p* < 0.001) compared to the delayed group. In the delayed DIEP group there were significant differences in histological type, Bloom Richardson grade, axillary treatment, tumor stage, lymph node stage and endocrine tumor status (all *p* < 0.001) compared to the immediate DIEP group. Consequently, significantly more patients in the delayed DIEP group received chemotherapy, endocrine therapy and radiation therapy. Furthermore, these patients more frequently had a history of implants or tissue expanders (*p* < 0.001). In the immediate DIEP flap group, patients more often had a history of lumpectomy (*p* < 0.001) and more frequently underwent surgery in the university hospital (*p* < 0.001) as compared to the delayed group.

**Table 1:** Patient demographics (*n* = 862 patients)

	Immediate DIEP *n* (%)	Delayed DIEP *n* (%)	*p* value
Total number of patients	326	536	
Total number of DIEP flaps	347	572	
Age in years; mean ± SD	52.79 ± 8.28	50.92 ± 8.48	< 0.001
BMI; mean ± SD	26.11 ± 3.72	26.95 ± 3.71	< 0.001
History of lumpectomy^a^	106 (30.5)	88 (15.4)	< 0.001
History of tissue expanders/implants^a^	4 (1.2)	166 (29.0)	< 0.001
Type of reconstruction			
Unilateral	244 (74.8)	411 (76.7)	< 0.001
Bilateral	76 (23.3)	108 (20.1)	
Stacked	6 (1.9)	17 (3.2)	
Side			
Left	179 (51.6)	293 (51.2)	0.845
Right	168 (48.4)	278 (48.6)	
Oncological treatment			
History of radiation therapy^a^	112 (32.3)	269 (47.0)	< 0.001
Chemotherapy	160 (49.1)	369 (68.8)	< 0.001
Endocrine therapy	136 (41.7)	294 (54.9)	< 0.001
Immunotherapy	43 (13.2)	79 (14.7)	0.065
Hospital setting			
University hospital	195 (59.8)	285 (53.2)	< 0.001
Community hospital	131 (40.2)	251 (46.8)	
Follow-up in months; median (IQR)^b^	46 (45)	56 (46)	< 0.001
Follow-up in months; median (IQR) ^c^	46 (45)	86 (54.75)	< 0.001

*DIEP* deep inferior epigastric artery perforator; *SD* standard deviation; *BMI* body mass index; *IQR* interquartile range

^a^Total number of flaps as unit of analysis (immediate group: *n* = 347; delayed group: *n* = 572)

^b^Time from breast reconstruction until last date of follow up or recurrence or death from another cause

^c^Time from mastectomy until last date of follow up or recurrence or death from another cause

**Table 2:** Tumor characteristics (*n* = 862 patients)

	Immediate DIEP *n* (%)	Delayed DIEP *n* (%)	*p* value
Total number of patients	326	536	
Total number of DIEP flaps	347	572	
Histological type (WHO classification)^a^			
DCIS	73 (22.4)	57 (10.6)	< 0.001
LCIS	1 (0.3)	0 (0)	
Invasive	115 (35.3)	229 (42.7)	
Invasive and DCIS	126 (38.7)	215 (40.1)	
Other	11 (3.4)	22 (4.1)	
Missing	0 (0)	13 (2.4)	
Bloom Richardson grade			
NA	2 (0.6)	0 (0)	< 0.001
1	50 (15.3)	80 (14.9)	
2	128 (39.3)	206 (38.4)	
3	122 (37.4)	156 (29.1)	
Missing	24 (7.4)	94 (17.5)	
Axillary treatment			
No treatment	13 (4.0)	11 (2.1)	< 0.001
SLN	252 (77.3)	236 (44.0)	
ALND	30 (9.2)	159 (29.7)	
SLN + ALND	29 (8.9)	116 (21.6)	
Missing	2 (0.6)	14 (2.6)	
Tumor stage^b^			
0	12 (3.7)	28 (5.2)	< 0.001
Tis	80 (24.5)	50 (9.3)	
1	132 (40.5)	167 (31.2)	
2	77 (23.6)	183 (34.1)	
3	7 (2.1)	47 (8.8)	
4	1 (0.3)	11 (2.1)	
Missing	17 (5.2)	50 (9.3)	
Y			
Yes	54 (16.6)	140 (26.1)	< 0.001
No	272 (83.4)	396 (73.9)	
Lymph Node stage^b^			
0	241 (73.9)	274 (51.1)	< 0.001
1	65 (19.9)	170 (31.7)	
2	6 (1.8)	54 (10.1)	
3	1 (0.3)	16 (3.0)	
Missing	13 (4.0)	22 (4.1)	
ER status			
NA	68 (20.9)	51 (9.5)	< 0.001
Positive	195 (59.8)	336 (62.7)	
Negative	59 (18.1)	96 (17.9)	
Missing	4 (1.2)	53 (9.9)	
PR status			
NA	68 (20.9)	51 (9.5)	< 0.001
Positive	154 (47.2)	273 (50.9)	
Negative	96 (29.4)	156 (29.1)	
Missing	8 (2.5)	56 (10.4)	
HER2 status			
NA	68 (20.9)	51 (9.5)	< 0.001
Amplified	53 (16.3)	87 (16.2)	
Not amplified	186 (57.1)	319 (59.5)	
Missing	19 (5.8)	79 (14.7)	

*DCIS* ductal carcinoma in situ; *LCIS* lobular carcinoma in situ; *NA* not applicable; *SLN* sentinel lymph node; *ALND* axillary lymph node dissection; *y* neoadjuvant therapy before mastectomy; *ER* estrogen receptor; *PR* progesterone receptor; *HER2* human epidermal growth factor receptor

^a^Histopathological primary tumor stage before receiving any therapy

^b^The initial pathology stage mentioned in the pathology record

### Recurrence rates

Specific recurrence rate details are summarized in Table 3. In the immediate reconstruction group, five patients (1.5%) experienced a local recurrence versus 9 patients (1.7%) in the delayed group (*p* = 0.400). Regional recurrence occurred in 12 (3.7%) versus 13 (2.4%) patients (*p* < 0.001) (immediate versus delayed). Without adjustment this result indicates a greater risk of regional recurrence in the immediate reconstruction group. In contrast, distant recurrence occurred in 2.8% of women in the immediate group versus 6.9% (*p* = 0.085) in the delayed group, indicating a greater but not significant risk of distant recurrence in the delayed reconstruction group. The median time between an immediate reconstruction and the first recurrence was 32.5 months compared to 26.5 months in the delayed reconstruction group.

**Table 3:** Recurrence rate details (*n* = 862 patients)

	Immediate DIEP *n* (%)	Delayed DIEP *n* (%)	HR (95% CI)	*p* value	Adjusted HR (95% CI)^a^	Adjusted *p* value ^a^
Total number of patients	326	536				
Total number of DIEP flaps	347	572				
Local recurrence	5 (1.5)	9 (1.7)	0.804 (0.483–1.337)	0.400	2.890 (1.536–5.437)	0.001
Regional recurrence	12 (3.7)	13 (2.4)	0.306 (0.214–0.436)	< 0.001	0.912 (0.627–1.327)	0.631
Total number of patients^b^	251	466				
Total number of DIEP flaps^b^	267	494				
Distant recurrence	7 (2.8)	32 (6.9)	1.351 (0.960–1.903)	0.085	5.244 (3.395–8.102)	< 0.001

^a^Adjusted for immortal time bias, age at operation, histological tumor type, TN stage, *y* stage, Bloom Richardson grade, axillary treatment, chemotherapy, radiation therapy, endocrine therapy and immunotherapy

^b^In the analysis for distant recurrence, in situ carcinomas were excluded

The hazard ratios of the univariable analyses with local recurrence as the primary endpoint were adjusted for potential confounding variables (i.e. immortal time bias, age at operation, histological tumor type, TN stage, *y* stage, Bloom Richardson grade, axillary treatment, chemotherapy, radiation therapy, endocrine therapy and immunotherapy) in multivariable models (Table 4). Patients with a age above 40 were significantly less likely to have a local recurrence than younger patients (HR 0.274; 95% CI 0.142–0.527; *p* < 0.001), even after adjustment (HR 0.044; 95% CI 0.016–0.121; *p* < 0.001). In addition, not receiving chemotherapy and radiotherapy, before (HR 2.976; 95% CI 1.801–4.918; *p* < 0.001) (HR 0.583; 95% CI 0.365–0.932; *p* = 0.024) and after adjustment (HR 7.404; 95% CI 3.146–17.425; *p* < 0.001) (HR 0.347; 95% CI 0.180–0.669; *p* = 0.001), significantly increased the risk of local recurrence. For some variables, the number of events was too low to validly estimate associations. Identical adjustments were performed in multivariable analysis of regional and distant recurrence. The adjusted hazard ratios of local recurrence (HR 2.890; 95% CI 1.536–5.437; *p* = 0.001) and distant recurrence (HR 5.244; 95% CI 3.395–8.102; *p* < 0.001) became significant. These results indicates a greater risk of developing a recurrence after delayed DIEP flap reconstruction surgery compared to an immediate reconstruction. Interestingly, the adjusted hazard ratio of regional recurrence (HR 0.912; 95% CI 0.627–1.327; *p* = 0.631) was no longer statistically significant. This indicates that at least part of the association was due to confounders instead of timing of breast reconstruction.

**Table 4:** Univariable and multivariable Cox regression analysis with local recurrence as the primary endpoint

	Univariable analysis		Multivariable analysis	
	HR (95% CI)	*p* value	HR (95% CI)^a^	*p* value
*DIEP*				
Immediate	1.00 (reference)		1.00 (reference)	
Delayed	0.804 (0.483–1.337)	0.400	2.890 (1.536–5.437)	0.001
*Age at operation*				
≤ 40	1.00 (reference)	< 0.001	1.00 (reference)	< 0.001
41–50	0.274 (0.142–0.527)	< 0.001	0.044 (0.016–0.121)	< 0.001
≥ 51	0.330 (0.187–0.582)		0.102 (0.045–0.232)	< 0.001
*Histological tumor type*				
In situ	1.00 (reference)^b^		1.00 (reference)^b^	
*Invasive*				
*T* stage				
0	1.00 (reference)^b^		1.00 (reference)^b^	
*In situ*				
1				
2				
3				
4				
*N* stage				
0	1.00 (reference)^b^		1.00 (reference)^b^	
1				
2				
3				
*y* stage				
Yes	1.00 (reference)		1.00 (reference)	
No	2.640 (1.141–6.110)	0.023	1.405 (0.496–3.980)	0.522
*Bloom Richardson grade*				
1	1.00 (reference)^b^		1.00 (reference)	
2			0.677 (0.349–1.314)	0.249
3			0.148 (0.049–0.448)	0.001
*Axillary treatment*				
No treatment	1.00 (reference)^b^		1.00 (reference)^b^	
SLN				
ALND				
SLN + ALND				
Chemotherapy				
Yes	1.00 (reference)		1.00 (reference)	
No	2.976 (1.801–4.918)	< 0.001	7.404 (3.146–17.425)	< 0.001
*Radiation therapy*				
Yes	1.00 (reference)		1.00 (reference)	
No	0.583 (0.365–0.932)	0.024	0.347 (0.180–0.669)	0.002
*Endocrine therapy*				
Yes	1.00 (reference)		1.00 (reference)	
No	1.098 (0.672–1.793)	0.710	1.835 (0.955–3.526)	0.068
*Immunotherapy*				
Yes	1.00 (reference)		1.00 reference	
No	1.628 (0.703–3.769)	0.256	1.036 (0.370–2.900)	0.947

*y* neoadjuvant therapy before mastectomy; *SLN* sentinel lymph node; *ALND* axillary lymph node dissection

^a^Adjusted for immortal time bias, age at operation, histological tumor type, TN stage, y stage, Bloom Richardson grade, axillary treatment, chemotherapy, radiation therapy, endocrine therapy and immunotherapy

^b^Too few events to validly estimate associations

Lastly, univariable and multivariable analyses of tumor characteristics and recurrence rate details with the flap as the unit of analysis showed comparable results (see additional data: “Online Resource 1: Table 1 and 2”).

## Discussion

We performed a multicenter retrospective cohort study to determine the rate of breast cancer recurrence in women who underwent an immediate as compared to a delayed autologous DIEP flap breast reconstruction. Our aim was to analyse the association in timing and breast cancer recurrence between immediate and delayed DIEP flap reconstruction.

The advantage of an immediate breast reconstruction is the one-stage reconstruction. A skin-sparing technique can be used which offers better cosmetic results, less distress and better self-esteem, satisfaction and body image compared to those who opt for delayed reconstruction [7, 16, 17]. The overall cost is less because only one operation is needed, lowering inpatient hospital days and operation time [18]. Nevertheless, there are also limitations. When postoperative complications occur there might be a potential delay for adjuvant therapy [19] Another negative factor, mentioned in the immediate setting, is that planning an operation in which a plastic surgeon and an oncological surgeon are present at the same time is often difficult [20]. Furthermore, this procedure is not suitable in case of the need for adjuvant radiotherapy, which would jeopardize the outcome of the reconstruction. In addition, postoperative radiation therapy is sometimes needed in residual disease or close surgical margins, which can adversely affect the aesthetic outcome [21, 22]. Ideally, the indication for postmastectomy adjuvant radiotherapy should be known preoperatively. This indication is based on patient and tumor characteristics. Combining preoperative imaging, biopsy and patient characteristics allows prediction of the chance that a woman might need adjuvant radiotherapy. When this chance is high, women are advised to opt for delayed reconstruction.

Limitations of delayed reconstruction include a poorer aesthetic result (no skin sparing mastectomy), higher costs to the health care system and prolonging the overall treatment [21].

Previously, we mentioned there is no statistical difference in major complications between both procedures. Therefore, this particular factor will not be important in decision-making [13]. In summary, both procedures have their advantages and limitations.

This study showed that the adjusted hazard ratio for immediate versus delayed DIEP flap reconstruction surgery was 2.890 (*p* = 0.001, 95% CI 1.536, 5437) for local breast cancer recurrence and 5.244; (*p* < 0.001, 95% CI 3.395–8.102) for distant recurrence. This indicates that in our study women who received a delayed reconstruction surgery would have an almost three to five times greater risk of developing a recurrence after DIEP flap reconstruction surgery than women who received an immediate reconstruction.

Previous studies have shown contradictory results regarding the effect of DIEP flap reconstruction on the breast cancer recurrence rate. A cohort study of Isern et al. reported an increased risk of breast cancer recurrence in the delayed autologous reconstruction group compared to mastectomy alone [12]. However, the statistical power in this study was limited. Only 33 DIEP flap reconstructions were included. Two Swedish studies described no difference in breast cancer recurrence between delayed DIEP flap reconstruction groups and mastectomy alone. A major limitation of these studies is that they included a relatively small number of DIEP flap reconstruction patients in a long period of time, 250 patients in a 14 year time period, and 225 patients in 9 years respectively [13, 14]. A study by Geers et al. assessed the effect of an (immediate or delayed) autologous breast reconstruction as compared to mastectomy alone, on distant relapse. In this study, 485 patients with autologous breast reconstruction did not show a higher risk of metastatic disease as compared to patients with mastectomy [15]. However, none of these studies focused on the specific effect of timing (immediate or delayed) on breast cancer recurrence in DIEP flap reconstruction surgery in a clinical setting. Therefore, our results cannot be compared directly to other studies and should be regarded as a first exploration in this field.

A study by Dillekas et al. characterized the recurrence pattern in breast cancer patients after delayed reconstruction (implant and/or autologous), as compared to patients without reconstruction. This study has shown that breast cancer relapse shows a peak at 2 years and at 5–6 years after mastectomy. In addition, when time origin is placed at the delayed breast reconstruction, a similar bipolar peak in recurrence rate appears [11]. The timing of the transition from dormant micro metastases into clinically detectable macro metastases might be explained by an enhancing effect of surgery, due to reactivation of dormant micro metastasis by releasing angiogenic agonists, growth factors and immunomodulating factors after repeated tissue trauma [9, 11, 23–32].

The hypothesis that breast cancer recurrence is associated with surgical interventions cannot be compared directly with our results because either immediate or delayed procedure might induce reactivation of dormant tumor cells by its major surgical trauma. Still, the results of Dillekas et al. shows indirect evidence delayed reconstruction would be less beneficial than immediate as this costs one more possible growth inducing event. However, only 28% of the study group comprised autologous flap reconstruction, which calls this statement into question [11]. Furthermore, with mentioned hypothesis the previous described studies should have shown more recurrences in the delayed reconstruction group compared to the mastectomy alone, which they did not (Isern et al. excluded) [13–15]. In addition, a study by Allawi et al. did not find any association between surgery or accidental trauma and activation of dormant micro metastases leading to higher recurrence rates of breast cancer [33]. Therefore, based on the current literature and the minimal evidence of the mentioned hypothesis, we cannot biologically explain the almost three times greater risk of a local breast cancer recurrence in our study.

Even though this hazard ratio is adjusted for several confounders as well as for immortal time bias, the possibility of a selection mechanism, beyond what can be explained by covariates in our cohort, remains. It is evident from our baseline table that women from both groups are different by any measure. The choice between an immediate or delayed breast reconstruction is determined by several clinical factors, as well as the patient’s preference and her (emotional) ability to make well-informed decisions at the time of mastectomy [34]. This introduces an important limitation in our study design. As the decision process for a woman to receive either immediate or delayed reconstruction surgery is complex and multidimensional, it is extremely hard to draw solid conclusions in any study design that would be observational rather than experimental. The possibility remains that women receiving a delayed reconstruction are significantly different from women receiving an immediate reconstruction in regard to factors that we are not aware of and/or have not accounted for. The phenomenon of ‘residual confounding’ should therefore be taken into account as a limitation of this study and caution should be given to the interpretation of the hazard ratios.

Another important limitation lies in the difference between women who never opt for a reconstruction and women who, after not having an immediate reconstruction, opt for a delayed reconstruction later in life. In this research, we only look at women who choose to have breast reconstructive surgery. Differences in characteristics influencing recurrence rate might exist between women ‘never having reconstruction’ and ‘having delayed reconstruction’. This scenario would, again, imply a special case of selection bias and would therefore unjustly allocate the influence on recurrence rate to the timing of the surgery, rather than to the characteristics actually causing the differences.

Ideally, a randomized controlled trial would be most suitable to avoid this bias. Obviously, ethical reasons make this impossible. As an alternative, future studies might benefit by the introduction of an additional ‘control group’, consisting of women not opting for reconstruction surgery. In that case, a prospective matched cohort study would be most suitable in which a large study sample is necessary.

Major strengths of this study include the long period of follow up (median follow up 68 months) and a strong statistical model that accounts for several types of bias among which immortal time bias. The other specific covariates for adjustment were chosen carefully based on literature search and clinical experience. By avoiding backward elimination, we have ensured that the study is methodologically sound and generalisable. Lastly, an important observation of this study is that per-flap analysis provided comparable results to the per-patient analysis.

## Conclusions

This study suggests an increased risk for breast cancer recurrence in women receiving a delayed DIEP flap reconstruction. However, the results should be interpreted carefully due to potential selection bias and the limitations inherent to its retrospective design. Further research with a larger sample size is needed to elaborate on the findings of this study and to provide recommendations for clinical practice.

## Electronic supplementary material

Below is the link to the electronic supplementary material.

Supplementary material 1 (DOCX 21 kb)

\

## References

[1] Litière S (2012). Breast conserving therapy versus mastectomy for stage I-II breast cancer: 20 year follow-up of the EORTC 10801 phase 3 randomised trial. Lancet Oncol.

[2] Fisher B (2002). Twenty-year follow-up of a randomized trial comparing total mastectomy, lumpectomy, and lumpectomy plus irradiation for the treatment of invasive breast cancer. N Engl J Med.

[3] McCahill LE (2009). Are mastectomy rates a reasonable quality measure of breast cancer surgery?. Am J Surg.

[4] Skraastad BK (2019). Quality of life, patient satisfaction and cosmetic outcome after delayed breast reconstruction using DIEP flap: a 10 years' follow-up survey. J Plast Surg Hand Surg.

[5] Cordeiro PG (2008). Breast reconstruction after surgery for breast cancer. N Engl J Med.

[6] Allen RJ, Treece P (1994). Deep inferior epigastric perforator flap for breast reconstruction. Ann Plast Surg.

[7] Zhong T (2016). A comparison of psychological response, body image, sexuality, and quality of life between immediate and delayed autologous tissue breast reconstruction: a prospective long-term outcome study. Plast Reconstr Surg.

[8] Beugels J (2018). Complications following immediate compared to delayed deep inferior epigastric artery perforator flap breast reconstructions. Breast Cancer Res Treat.

[9] Demicheli R (2008). Recurrence dynamics does not depend on the recurrence site. Breast Cancer Res.

[10] Curigliano G (2005). Systemic effects of surgery: quantitative analysis of circulating basic fibroblast growth factor (bFGF), vascular endothelial growth factor (VEGF) and transforming growth factor beta (TGF-β) in patients with breast cancer who underwent limited or extended surgery. Breast Cancer Res Treat.

[11] Dillekås H (2016). The recurrence pattern following delayed breast reconstruction after mastectomy for breast cancer suggests a systemic effect of surgery on occult dormant micrometastases. Breast Cancer Res Treat.

[12] Isern A (2011). Risk of recurrence following delayed large flap reconstruction after mastectomy for breast cancer. Br J Surg.

[13] Adam H (2018). Risk of recurrence and death in patients with breast cancer after delayed deep inferior epigastric perforator flap reconstruction. Br J Surg.

[14] Svee A (2018). Survival and risk of breast cancer recurrence after breast reconstruction with deep inferior epigastric perforator flap. Br J Surg.

[15] Geers J (2018). Oncological safety of autologous breast reconstruction after mastectomy for invasive breast cancer. BMC Cancer.

[16] Neyt M (2005). Comparing the cost of delayed and immediate autologous breast reconstruction in Belgium. Br J Plast Surg.

[17] Al-Ghazal S (2000). The psychological impact of immediate rather than delayed breast reconstruction. Eur J Surg Oncol.

[18] Khoo A (1998). A comparison of resource costs of immediate and delayed breast reconstruction. Plast Reconstr Surg.

[19] Newman LA (1998). Presentation, treatment, and outcome of local recurrence after skin-sparing mastectomy and immediate breast reconstruction. Ann Surg Oncol.

[20] Munhoz AM (2007). Immediate skin-sparing mastectomy reconstruction with deep inferior epigastric perforator (DIEP) flap. Technical aspects and outcome. Breast J.

[21] Hu E, Alderman AK (2007). Breast reconstruction. Surg Clin North Am.

[22] Ananthakrishnan P, Lucas A (2008). Options and considerations in the timing of breast reconstruction after mastectomy. Clevel Clin J Med.

[23] Ghajar CM (2013). The perivascular niche regulates breast tumour dormancy. Nat Cell Biol.

[24] Luzzi KJ (1998). Multistep nature of metastatic inefficiency: dormancy of solitary cells after successful extravasation and limited survival of early micrometastases. Am J Pathol.

[25] Neeman E, Ben-Eliyahu S (2013). Surgery and stress promote cancer metastasis: new outlooks on perioperative mediating mechanisms and immune involvement. Brain Behav Immun.

[26] Demicheli R (2005). Breast cancer recurrence dynamics following adjuvant CMF is consistent with tumor dormancy and mastectomy-driven acceleration of the metastatic process. Ann Oncol.

[27] Demicheli R (2007). Tumor dormancy and surgery-driven interruption of dormancy in breast cancer: learning from failures. Nat Clin Pract Oncol.

[28] Retsky MW (2008). Dormancy and surgery-driven escape from dormancy help explain some clinical features of breast cancer. APMIS.

[29] Goldfarb Y, Ben-Eliyahu S (2007). Surgery as a risk factor for breast cancer recurrence and metastasis: mediating mechanisms and clinical prophylactic approaches. Breast Dis.

[30] Tsuchiya Y (2003). Increased surgical stress promotes tumor metastasis. Surgery.

[31] Ben-Eliyahu S (2003). The promotion of tumor metastasis by surgery and stress: immunological basis and implications for psychoneuroimmunology. Brain Behav Immun.

[32] Ogawa K (2000). Suppression of cellular immunity by surgical stress. Surgery.

[33] Allawi Z, Cuzick J, Baum M (2012). Does trauma or an intercurrent surgical intervention lead to a short-term increase in breast cancer recurrence rates?. Ann Oncol.

[34] Yoon AP (2018). Outcomes of immediate versus delayed breast reconstruction: results of a multicenter prospective study. The Breast.

