# Supplementary material for: The difference in local, regional and distant breast cancer recurrence between the immediate and delayed DIEP flap procedure; a retrospective cohort study
Source: Breast Cancer Res Treat. 2021 May 24;188(2):389–98. doi: 10.1007/s10549-021-06199-3 (PMC8260410; doi:10.1007/s10549-021-06199-3)
Supplement: Supplementary file 1 — Supplementary material 1 (DOCX 21 kb) [file 10549_2021_6199_MOESM1_ESM.docx]

**Additional data**

**Table 1** Tumor characteristics (n = 919 DIEP flap breast reconstruction procedures)

|  | **Immediate**  **DIEP**  **n (%)** | **Delayed**  **DIEP**  **n (%)** | ***p* value** |
| --- | --- | --- | --- |
| Total number of patients | 326 | 536 |  |
| Total number of DIEP flaps | 347 | 572 |  |
|  |  |  |  |
| Histological type (WHO classification)^a^ |  |  |  |
| DCIS | 78 (22.5) | 64 (11.2) | <0.001 |
| LCIS | 1 (0.3) | 0 (0) |  |
| Invasive | 121 (34.9) | 244 (42.7) |  |
| Invasive and DCIS | 134 (38.6) | 227 (39.7) |  |
| Other  Missing | 13 (3.7)  0 (0) | 22 (3.8)  15 (2.6) |  |
| Bloom Richardson grade |  |  |  |
| NA | 2 (0.6) | 0 (0) | <0.001 |
| 1 | 56 (16.1) | 90 (15.7) |  |
| 2 | 134 (38.6) | 222 (38.8) |  |
| 3  Missing | 129 (37.2)  26 (7.5) | 160 (28.0)  100 (17.5) |  |
| Axillary treatment |  |  |  |
| No Treatment | 14 (4.0) | 11 (1.9) | <0.001 |
| SLN | 271 (78.1) | 258 (45.1) |  |
| ALND | 31 (8.9) | 167 (29.2) |  |
| SLN + ALND  Missing | 29 (8.4)  2 (0.6) | 120 (21.0)  16 (2.8) |  |
| Tumor stage^b^ |  |  |  |
| 0 | 14 (4.0) | 29 (5.1) | <0.001 |
| Tis | 85 (24.5) | 57 (10.0) |  |
| 1 | 144 (41.5) | 177 (30.9) |  |
| 2 | 77 (22.2) | 196 (34.3) |  |
| 3 | 7 (2.0) | 49 (8.6) |  |
| 4  Missing | 1 (0.3)  19 (5.5) | 11 (1.9)  53 (9.3) |  |
| y  Yes  No | 59 (17.0)  288 (83.0) | 150 (26.2)  422 (73.8) | <0.001 |
| Lymph Node stage^b^ |  |  |  |
| 0 | 260 (74.9) | 296 (51.7) | <0.001 |
| 1 | 67 (19.3) | 178 (31.1) |  |
| 2 | 6 (1.7) | 57 (10.0) |  |
| 3  Missing | 1 (0.3)  13 (3.7) | 17 (3.0)  24 (4.2) |  |
| ER status |  |  |  |
| NA | 72 (20.7) | 58 (10.1) | <0.001 |
| Positive | 210 (60.5) | 355 (62.1) |  |
| Negative  Missing | 61 (17.6)  4 (1.2) | 98 (17.1)  61 (10.7) |  |
| PR status |  |  |  |
| NA | 72 (20.7) | 58 (10.1) | <0.001 |
| Positive | 169 (48.7) | 288 (50.3) |  |
| Negative  Missing | 98 (28.2)  8 (2.3) | 162 (28.3)  64 (11.2) |  |
| HER2 status |  |  |  |
| NA | 72 (20.7) | 59 (10.3) | <0.001 |
| Amplified | 55 (15.9) | 89 (15.6) |  |
| Not amplified  Missing | 200 (57.6)  20 (5.8) | 338 (59.1)  86 (15.0) |  |

DCIS, ductal carcinoma in situ; LCIS, lobular carcinoma in situ; NA, not applicable; SLN, sentinel lymph node; ALND, axillary lymph node dissection; y, neoadjuvant therapy before mastectomy; ER, estrogen receptor; PR, progesterone receptor; HER2, human epidermal growth factor receptor. ^a^ Histopathological primary tumor stage before receiving any therapy. ^b^The initial pathology stage mentioned in the pathology record.

**Table 2** Recurrence rate details (n = 919 DIEP flap breast reconstruction procedures)

|  | **Immediate DIEP**  **n (%)** | **Delayed**  **DIEP**  **n (%)** | **HR**  **(95% CI)** | ***p* value** | | **Adjusted HR**  **(95% CI)^a^** | **Adjusted *p* value ^a^** |
| --- | --- | --- | --- | --- | --- | --- | --- |
| Total number of patients | 326 | 536 |  |  |  | |  |
| Total number of DIEP flaps | 347 | 572 |  |  |  | |  |
|  |  |  |  |  |  | |  |
| Local recurrence | 5 (1.4) | 9 (1.6) | 0.796 (0.478 – 1.326) | 0.382 | 2.897 (1.539–5.452) | | 0.001 |
| Regional recurrence | 12 (3.5) | 13 (2.3) | 0.301 (0.211 – 0.430) | <0.001 | 0.892 (0.614–1.296) | | 0.549 |
|  |  |  |  |  |  | |  |
| Total number of patients^b^ | 251 | 466 |  |  |  | |  |
| Total number of DIEP flaps^b^ | 267 | 494 |  |  |  | |  |
|  |  |  |  |  |  | |  |
| Distant recurrence | 7 (2.6) | 32 (6.5) | 1.356 (0.963-1.910) | 0.081 | 4.980 (3.249–7.634) | | <0.001 |

^a^Adjusted for immortal time bias, age at operation, histological tumor type, TN stage, y stage, Bloom Richardson grade, axillary treatment, chemotherapy, radiation therapy, endocrine therapy and immunotherapy

^b^In the analysis for distant recurrence, in situ carcinomas were excluded
